# Insights into the Interplay between the Urinary Microbiome and Bladder Cancer: A Comprehensive Review

**DOI:** 10.3390/jcm13164927

**Published:** 2024-08-21

**Authors:** Rigoberto Pallares-Mendez, Aldo Brassetti, Alfredo Maria Bove, Giuseppe Simone

**Affiliations:** Department of Urology, IRCCS Regina Elena National Cancer Institute, Via Elio Chianesi, 53, 00128 Rome, Italy; rigo_pallares@hotmail.com (R.P.-M.);

**Keywords:** microbiota, virome, urinary bladder neoplasms, non-muscle-invasive bladder neoplasms, *Mycobacterium bovis*

## Abstract

New insights in the urinary microbiome have led to a better understanding being built of the shifts in bacterial representations from health to disease; these hold promise as markers for diagnosis and therapeutic responses. Although several efforts have been made to identify a “core urinary microbiome”, different fingerprints have been identified in men and women that shift with age. The main bacterial groups overall include *Firmicutes*, *Actinobacteria*, *Fusobacteria*, and *Bacteroidetes*. Although patients with bladder cancer have a microbiome that is similar to that of healthy individuals, differences have been observed at the species level with *Fusobacterium nucleatum* and *Ralstonia*, and at the genus level with *Cutibacterium*. Different bacterial representations may influence extracellular matrix composition, affecting tumor metastatic spreading and tumorigenic metalloproteinase expression. Furthermore, gene expression affecting targets of immune therapy, such as PD-L1, has been associated with changes in bacterial representations and therapeutic response to BCG. This comprehensive review aims to examine the influence of the urinary microbiome in bladder cancer.

## 1. Introduction

The urinary microbiome plays an important role in maintaining the urinary bladder’s microenvironment, influencing both health and disease. The urinary microbiome varies with age and sex [1]; furthermore, its disruption may cause inflammatory pathway activation that could potentially lead to the development of bladder cancer [2].

Research on the influence of the urinary microbiome in bladder cancer holds promise in identifying biomarkers for diagnosis, prognosis, and therapeutic response. Additionally, targeting or modifying bacterial populations may become a future therapeutic strategy in treating bladder cancer [3]. In this review, we examine the influence of the urinary microbiome on the vesical microenvironment, its role in promoting carcinogenic pathways, and future perspectives on its applications.

## 2. Methods

The PICO framework was employed to ensure an effective search process. The population included men and women diagnosed with non-invasive or muscle-invasive bladder cancer, as well as those undergoing Bacillus Calmette–Guérin (BCG) therapy or other immunotherapies. The intervention involved measuring microbiome representations using various molecular biology techniques, such as 16S or 18S rRNA sequencing, whole-metagenome sequencing, shotgun metagenome sequencing, 16S-ITS amplicon sequencing, and extended quantitative urine culture. Comparisons were conducted using control groups. The evaluated primary outcomes were alpha and beta diversity, as well as bacterial representation percentages in patients with bladder cancer compared to controls. Secondary outcomes included assessing these same parameters in patients undergoing BCG therapy or other systemic therapies.

A literature search was conducted with PubMed and Scopus from January 2024 to July 2024. Studies were included if they investigated the role of the urinary microbiome in bladder cancer, involved human or animal models, were published in English, and were observational studies, clinical trials, case–control studies, or meta-analyses. Studies with insufficient data or from non-peer-reviewed sources were excluded.

Search entries were introduced as a combination of (“Bladder cancer” OR “BCG Therapy” OR “BCG Response” OR “Bacillus Calmette-Guerin”) AND (“Microbiome” OR “Urobiome” OR “Urinary Microbiome”) OR a combination of (“Bladder Cancer”) AND (“Systemic Therapy” OR “Chemotherapy”) AND (“Microbiome” OR “Urobiome” OR “Urinary Microbiome”). The literature search showed 312 papers; from these, 225 were excluded after deduplication. After article screening and full-text reading, a total of 32 analytical comparative studies were included for discussion, with the remaining studies listed in the references as supporting evidence.

## 3. Revisiting the “Core” Urinary Microbiome

Seminal studies on the urinary bladder’s microbiome, analyzed through 16S rRNA PCR sequencing, have shown distinct bacterial representations. In men, the primary bacterial phyla include *Firmicutes* (52.6%), *Actinobacteria* (18.7%), *Fusobacteria* (10%), *Proteobacteria* (9.4%), and *Bacteroidetes* (7.4%). At the genus level, *Lactobacillus*, *Corynebacterium*, and *Streptococcus* were observed in up to 50% of the urinary samples [4].

In contrast, the female urinary microbiome displays a slightly different bacterial composition: *Firmicutes* (65%), *Bacteroidetes* (18%), *Actinobacteria* (12%), *Fusobacteria* (3%), and *Proteobacteria* (2%) [5]. Although more phyla have been identified in the female urinary microbiome, their representation does not exceed 1%. Different from the male urinary microbiome, analysis at the genus level showed a predominance of *Lactobacillus*, *Prevotella*, and *Gardnerella* [5].

The urinary microbiome is dynamic, with constant change in its bacterial representations through aging [6]. While *Firmicutes* remains the predominant phylum in both men and women, the latter exhibit higher bacterial diversity compared to men, displaying a higher abundance of *Actinobacteria* and *Bacteroidetes*. Furthermore, older women show a decrease in bacterial diversity compared to younger women [7,8]. 

Pearce and colleagues described a variety of six different urotypes based on the most dominant genus types: (1) *Gardnerella*, (2) *Sneathia*, (3) *Staphylococcus*, (4) *Enterobacteriaceae*, (5) *Lactobacillus*, and (6) a “diverse” urotype with an even distribution of genera. Among patients without bladder cancer, *Lactobacillus* is the most common urotype (60%), followed by *Gardnerella* (12%) [8].

## 4. The Urinary Microbiome in Bladder Cancer

Determining whether a microbiome dysbiosis precedes or proceeds bladder cancer is yet to be studied. Several efforts have been made to address the role of the urinary microbiome in bladder cancer. Bucevic Popovic et al. [9] observed no significant differences in overall microbiome diversity at the phylum or family level. However, significant differences were observed regarding operational taxonomic units (OTUs) enriched in patients with bladder cancer, including *Fusobacteria*, *Actinobacteria*, *Facklamia*, and *Campylobacter*. Specific OTUs identified at the strain level were *Campylobacter hominis*, *Actinobaculum massiliense*, and *Jonquetella antrophi.* Further analysis highlighted *Fusobacterium nucleatum* as a protumorigenic pathogen, which was detected in 26% of bladder cancer tissues analyzed using polymerase chain reaction (PCR).

One study performed by Chorbinska et al. [10] observed no differences in alpha or beta diversity in the urinary microbiome between patients with bladder cancer and controls without bladder cancer when midstream urine samples were analyzed. The most predominant phyla in both groups were *Firmicutes*, *Proteobacteria*, and *Actinobacteria. Lactobacillus* was more common in patients with a history of BCG therapy. Similarly, Hussein et al. [11] observed no differences in alpha diversity but noted contrasting results in beta diversity, with greater representation at a phylum level of *Firmicutes* and *Deinococcus* in controls and *Actinomyces* among other bacteria in patients with bladder cancer. These observations are consistent with those of Bi et al. [12], who studied 29 patients with bladder cancer and 26 controls.

Differences in urinary microbiome composition were elegantly assessed in a study of 22 patients, including 7 with NMIBC and 15 with MIBC [13]. The analysis involved DNA extraction from bladder tissue samples and comparison of DNA reads using type IIB restriction endonuclease, categorizing the microbiome up to the species level. This study showed 165 species shared between NMIBC and MIBC, while 265 species were unique to the MIBC group. Significant differences in alpha diversity, measured by the Shannon and Simpson indexes, indicated greater richness and diversity in NMIBC. Beta diversity was also different between groups. Analysis at the phylum level in both MIBC and NMIBC showed a major representation of *Proteobacteria* (68.45% and 39.09%), *Firmicutes* (14.76% and 19.17%), and *Actinobacteria* (12.98% and 14.92%, respectively). However, when assessing differential abundance at the phylum level, *Proteobacteria* were increased in MIBC, while *Bacteroidetes* and *Firmicutes* were decreased. At a more specific level, this study identified *Ralstonia* and *Cutibacterium* as key genera in distinguishing MIBC from NMIBC, with an AUC of 95.2%. These findings, however, need to be validated in further prospective studies.

Consistent results were observed by Wu et al. [14], who observed four dominant phyla: *Proteobacteria*, *Firmicutes*, *Actinobacteria*, and *Bacteroidetes*; however, there were no significant differences between cancer and noncancer subjects at the phylum level. At the genus level, significant differences were observed with *Acinetobacter*, which was more abundant in patients with bladder cancer.

Another study, which included both men and women with bladder cancer (either MIBC or NMIBC) and controls without bladder cancer with benign urologic conditions, analyzed the microbiome in midstream samples. Results showed a higher diversity and richness in control samples when compared to those from patients with NMIBC and MIBC. These findings were consistent when comparing control samples with high-grade and low-grade bladder cancer samples. Taxa such as *Bacteroidetes*, *Lachnoclostridium*, and *Burkholderia* were predominantly represented in control samples, while MIBC showed a major representation of *Bacteroidetes* [15].

The inconsistency of results across studies is noteworthy. Diverse observations on the “core” urinary microbiome in bladder cancer may be affected by several methodological variations. These include inclusion criteria that analyze both men and women together [7,8,16], the inclusion of both muscle-invasive bladder cancer and non-muscle-invasive bladder cancer [13], and a wide age range among individuals [6,7]. The evaluation of other independent variables such as diet, medication intake, or comorbidities was not addressed across the discussed studies. Although Wu et al. [14] evaluated comorbidities such as hypertension, diabetes, hyperlipidemia, and cardiac disease, observing no difference in frequency between cancer patients and healthy subjects, no sub-analyses were performed to evaluate the roles of these factors as modifiers in the urinary microbiome. Other variables that may impact the urinary microbiome include the grade of urothelial cancer and exposure to BCG therapy. Furthermore, the heterogeneity of the method of obtaining the analyzed samples, such as midstream clean-catch urine collection, urinary-catheter-obtained samples, and bladder-tissue-extracted samples [17], along with different DNA extraction and library identification, may play an important role in obtaining diverse results (Table 1).

## 5. Microbiome Influence on the Extracellular Matrix

The extracellular environment contains factors that can promote both health and disease. Disruption of this microenvironment can lead to tumor growth, spread of protumorigenic factors, and metastasis through tumor cell spreading and seeding [2]. The extracellular matrix (ECM) acts as a complex system incorporating cytokines, growth factors, proteins, and proteoglycans, whose interactions and components contribute to molecular ensembles that modulate health and disease [2].

In a prospective study of 125 patients, including 19 females, components of the extracellular matrix were analyzed [13]. Bladder tissue analysis revealed a higher expression of the extracellular matrix metalloproteinase inducer (EMMPRIN, also known as CD147 or Basigin) in squamous cell carcinoma compared to transitional cell carcinoma. Furthermore, EMMPRIN expression was associated with greater tumor invasiveness. Non-muscle-invasive transitional carcinomas showed negative or low EMMPRIN expression compared to MIBC. Similar observations were made with fascin immunostaining, which also showed a direct correlation with MIBC. EMMPRIN promotes tumor progression by inducing invasion and metastasis through the production of MMPs [19,20]. Fascin, a protein linked to actin cytoskeleton rearrangements, and associated with invasion and metastasis potential, was overexpressed, potentially disrupting epithelial junctions [13]. 

The susceptibility of the basement membrane to degradation by metalloproteinases plays a crucial role in tumor invasion and distant metastasis. Although metalloproteinases perform various physiological functions, their downregulation affects the ECM balance, leading to disease. Several MMPs have been identified in bladder cancer samples, including MMP-2, MT1-MMP, MMP-28, and, to a lesser degree, but still importantly, MMP-7, -9, -10, and -13, among others. Regarding tumor grade, MMP-1, -2, -8, and -10, among others, were identified in muscle-invasive disease [20].

Available data on the microbiome–ECM interaction have been indirectly observed from studies evaluating interactions between the gut microbiome and the ECM. These observations suggest that gut microbiome dysbiosis could promote an inflammatory microenvironment, potentially leading to fibrosis via TGF-ß [21]. This has led to the consideration that bacterial components in the bladder, similar to those in the colon, could cross the urothelial barrier and trigger inflammation and fibrosis [2]. However, there is still a lack of evidence directly addressing the influence of urinary microbiome dysbiosis on interactions that promote bladder cancer.

## 6. Microbiome Influence on Gene Expression

Efforts to understand how the microbiome influences gene expression in urothelial bladder tissue have been addressed by Chen et al. [22]. In a study comparing patients with NMIBC who were PD-L1-positive (n = 9) to those who were PD-L1-negative (n = 19), it was observed that PD-L1+ patients showed higher bacterial richness in alpha diversity (ACE index) and beta diversity (UniFrac distance). Furthermore, differences in bacterial representations were observed between PD-L1+ and PD-L1- patients, with an increase in *Fusofacteria*, *Proteobacteria*, and *Actinomyces*, as well as a decrease in *Bacteroidetes* in PD-L1+ patients; the latter showed a statistically significant difference. 

At the genus level, *Leptotrichia* showed higher representation in PD-L1+ patients compared to PD-L1- patients [22]. Several associations have been observed between *Leptotrichia* and proinflammatory cytosines, such as IL-6 and INFγ, which promote PD-L1 expression, which is an important marker of tumor inhibition of T-cell activity and immune system evasion [23]. Although causality has not yet been established, these proposed mechanisms are important for understanding tumor aggressiveness. Further prospective studies are needed to evaluate these correlational findings.

The influence of the urinary microbiome on gene expression, particularly on genes promoting bladder cancer, remains a fertile field to explore. Further *omics* studies addressing the interaction and the microenvironment regulation in health and disease could help improve diagnosis and targeted therapies for bladder cancer. Small but significant steps have been made to analyze the association between the urinary microbiome on gene expression. An analysis comparing germ-free (GF) mice and specific pathogen-free (SPF) mice showed clear differences in mice with a suppressed microbiota, affecting genes and several pathways involved in extracellular matrix and neuromuscular synaptic transmission. This suggests a pivotal role of the microbiome in gene expression and bladder function [24].

## 7. Microbiome Modulation through Bacillus Calmette–Guérin (BCG) Instillation Therapy

The mechanism by which BCG helps in the treatment of NMIBC is not yet fully understood. However, it is recognized that BCG modulates the immune system, which in turn eliminates tumor cells in the urothelium, improving both recurrence and progression. In a study involving fresh tumor samples, urine samples, and formalin-fixed paraffin-embedded (FFBE) blocks, two groups were identified: BCG responders (defined as having a minimum of 24 months free of disease) and non-BCG responders. Significant differences were observed in the microbiome between responders and non-responders; both bacterial richness and composition varied between groups, with *Lactobacillus* spp. showing a higher representation in BCG responders [25].

In a study conducted by Heidrich et al. [26], catheterized urine samples from males diagnosed with intermediate- or high-risk NMIBC were compared with control males with benign prostate enlargement (BPE). Catheterized urine samples were also collected before the first BCG, as well as before the fifth and sixth BCG instillations for induction and first of maintenance. No differences were observed between NMIBC patients and the control group in this study. Furthermore, no correlation was observed between bladder microbiome composition and stage, multifocality, or tumor size. Consistent with other studies [25,27], an enrichment of *Lactobacillus*, *Streptococcus*, and *Cutibacterium* was associated with a favorable treatment response after a first cystoscopy three months after the last instillation of BCG [26]. Follow-up after maintenance therapy showed no evidence of BCG integration to the bladder microbiome, with amplicon sequence variants of *Mycobacterium bovis* detected in only 9% of samples, with a detectable microbiome during BCG maintenance. A drawback of this study is that controls without bladder cancer were taken from patients with BPE, despite evidence indicating a relationship between the microbiome composition and LUTS [28]. 

Although some studies [25,26,27] have consistently observed an association between *Lactobacillus* and treatment response, other studies have reported conflicting results regarding shifts in *Lactobacillus* representation after BCG therapy [29]. In one study, urine collected via catheterization before each weekly instillation of BCG induction therapy and before a three-month follow-up cystoscopy was analyzed using 16S rRNA gene sequencing; they observed that a higher representation of *Aerococcus* was associated with higher recurrence rates. Additionally, some *Proteobacteria* genera, such as *Escherichia/Shigella* and *Ureaplasma*, were observed in patients who did not experience recurrence. Bacterial richness has been observed to decrease after BCG therapy, suggesting an immune-mediated mechanism post-instillation. A drawback of this study was the inclusion of both males and females [29]. 

In summary, several studies have identified BCG as an agent of change in the urinary microbiome. While differences in the urinary bacterial representations have not been observed before BCG therapy, shifts have been noted after BCG induction and during maintenance regarding an increase in *Lactobacillus* (Firmicutes) and some Proteobacteria. Identifying these genera could be useful in predicting BCG treatment response and recurrence, potentially aiding in clinical decision making. A future prospective clinical trial is awaited to assess the interactions between BCG and the bladder microbiome among patients with NMIBC [30] (Table 2).

## 8. Overlooked Components of the Urinary Microbiome and Its Implications on Therapeutic Responses

The urinary virome, often overlooked in studies of urinary microbiome homeostasis, includes bacteriophages and eukaryotic viruses as predominant components. In a study comparing patients with bladder cancer to controls using real-time PCR to assess DNA sequences, several viruses were identified: HCMV (2%), EBV (6%), HHV-6 (12.5%), HPV (15.2%), and BKPyV (15.5%). These were observed in varying proportions among specimens. Notably, Torque Teno Virus (TTV) had a higher viruria rate of 44.2%, and JC polyomavirus was present in 47.6% of samples. When comparing patients with bladder cancer to controls, the former group showed a higher abundance of HPV (24.5% vs. 4.3%, respectively) and TTV (53.6% vs. 33%, respectively); however, TTV differences did not remain significant after adjusting for age and gender [31].

The hypothesized role of HPV in bladder cancer involves the release of reactive oxygen species (ROS) and reactive nitrogen species (RNS), leading to DNA mutations, particularly affecting p53. Furthermore, the E6 protein of HPV may promote upregulation of APOBEC3B, a protein that deaminates cytidine to uridine, which in turn promotes DNA mutations. APOBEC3B has been implicated in urothelial cancer due to its role in increasing mutation burden and activating oncogenes. Additionally, it may enhance antiviral defense by inhibiting viral replication and assisting the immune system in fighting infections [32,33].

Another component of the microbiome is the mycobiome. A study evaluated 52 fecal samples for the gut mycobiome, comparing patients with bladder cancer and controls without bladder cancer, evaluating compositional differences regarding response to neoadjuvant chemotherapy. Fungal composition showed *Saccharomycetales* as the dominant fungal representation in controls without bladder cancer compared to patients with bladder cancer (95.27% vs. 48.99%), followed by *Hypocreales*. The interaction between chemotherapy (gemcitabine–cisplatin) and mycobiome composition and the abundances within the gut were assessed. An increased representation of *Hypocreales* and decreased representation of *Saccharomycetales* were observed in chemotherapy responders compared to non-responders (21.12% vs. 0.12%, and 50.34% vs. 78.30%, respectively). Furthermore, a decrease in mycobiome richness was observed in non-responders; although this did not reach statistical significance (*p* = 0.34), a clear difference in alpha diversity was observed (0.26 vs. 0.83). This study suggests that the gut mycobiome may play an important role in modulating tumor response to chemotherapy in patients with bladder cancer, indicating that a favorable and diverse mycobiome with decreased *Saccharomycetales* representation may favor immune response to chemotherapy [34].

## 9. Metabolic Implications of the Microbiome in Bladder Cancer

Although most studies evaluate microbiome compositions as a snapshot in time, it is crucial to understand how changes in the microbiome composition translate in a dynamic context. While few studies have addressed this issue in the urinary microbiome, important data exist regarding animal models and gut microbiome–bladder cancer interactions that should be discussed. In a study conducted on mice inoculated with MBT2 urinary bladder cells [35], two groups of interest were compared: mice treated with probiotics, gemcitabine, and cisplatin and mice treated with only gemcitabine and cisplatin. A metabolic analysis using PICRUSt2 assessed 1418 genes, of which 2.9% (n = 42) were statistically different between these two groups (*p* ≤ 0.05), including 35 upregulated and 7 downregulated genes in the group combining chemotherapy with probiotics. 

Observed metabolic pathways included the methyltransferase family of enzymes (precorrin-3B C(17)-methyltransferase, precorrin-4 C(17)-methyltransferase, cobalt-precorrin-4-methyltransferase, and cobalt-precorrin-5B C(1)-methyltransferase) and two upregulated enzymes related to SCFAs metabolism (EC:2.8.3.8, acetate CoA-transferase, and EC:2.8.3.9, butyrate-acetoacetate CoA-transferase) in the group combining probiotics. Short-chain fatty acids (SCFAs) are known to be important signal modulators in gut–host–tumor crosstalk, while methyltransferases play a key role in methylating substrates of the epigenome, influencing enzymes associated with gene regulation, such as DNA methyltransferases, histone acetyltransferases, and deacetylases. The involvement of these metabolic pathways was related with a decreased tumor volume after 12 days of examination (244 ± 142 mm^3^ for the probiotics + GC group vs. 355 ± 103 mm^3^ for the GC-only group). This study showed a beneficial effect in tumor treatment when probiotics were conjointly administered [35].

To understand the metabolic implications of the fungal communities in bladder cancer, metagenomic analyses were performed by PICRUSt using ITS1/2 rRNA amplicon data. KEGG modules were identified across bladder cancer samples through the analysis of KEGG orthology and module data. Shifts in functional distributions were observed, including increased expression of chitinase, beta-*N*-acetylhexosaminidase, pyruvate ferredoxin, dihydrolipoamide dehydrogenase, and UTP-1-Glucose-1-Phosphate uridylyltransferase, most of which are implicated in carbohydrate and amino acid metabolism [34].

Another study analyzed the fecal samples of patients with bladder cancer before surgery and compared them to samples of individuals without bladder cancer. A total of 32 patients with bladder cancer were compared to 15 controls [36]. Functional characterization of the gut microbiota revealed dysbiosis in patients with bladder cancer, affecting several pathophysiological pathways. Notable differences in metabolites between the two groups included reduced levels of 11Z-eicosenoic acid, 3-methoxy tyrosine, abrine, aniline-2-sulfonate, arachidic acid, conjugated linoleic acids, elaidic acid, glycylleucine, glycylproline, leucyl-glycine, linoelaidic acid, linoleic acid, nicotamine hypoxanthine dinucleotide, oleic acid, petroselinic acid, and ricinoleic acid, with only cholesterol sulfate being increased in patients with bladder cancer. These metabolites were correlated with specific gut bacterial representations, such as Bacteroidetes salyersiae, *Bacteroidetes* spp., and Bacteroidetes fragilis, among other Bacteroidetes. The observed metabolomic alterations are significant for butyrate-producing bacteria, which were reduced in patients with bladder cancer. These bacteria are known to promote probiotic restoration and balance in gut microbiota. Additionally, several metabolites play roles in tumorigenic progression, apoptosis, cell proliferation, local inflammation (e.g., secondary bile acids, deoxycholic acid, lithocholic acid), and immune response [36]. This is consistent with the findings of Bukavina et al. [34], who observed that SCFAs dysregulation in the gut microbiome affects energy supply to enterocytes and G-protein compound function, which in turn impacts histone deacetylase activity, promoting protumorigenic epigenetic changes.

Metagenomic and metatranscriptomic analyses of the urinary microbiome are currently lacking, representing a promising area of research for building an understanding of the dynamic interactions between the urinary microbiome and bladder cancer. Although most of the studies discussed here focus on the gut microbiome, changes in the urinary microbiome could similarly affect local and systemic metabolic responses, potentially promoting tumor progression and recurrence. Seminal studies that assess the metabolic pathways influenced by the urinary microbiome have identified changes in the glycerolipid metabolism, retinol metabolism, carotenoid biosynthesis, ABC transporters, and ECM1/ERK1/2 phosphorylation/MMP9, which are pathways that may be related to bladder cancer through their effect on cell membrane function and integrity, cell proliferation, DNA damage through oxidative stress, and cell signaling [13,14,24].

## 10. Discussion

The emerging techniques in next-generation sequencing (NGS) have allowed the collection of information regarding the urinary microbiome. “Shotgun” NGS enables comprehensive metagenomic analysis by sequencing DNA from multiple bacterial species. Conversely, RNASeq analyzes the transcriptome, offering dynamic insights into how bacterial community behavior within the microbiome changes from health to disease [37].

The urinary microbiome may surely interact with other system microbiomes, such as those in the gastrointestinal tract, which play crucial roles in metabolic processes. Locally produced metabolites may also affect systemic functions through absorption and transport via the bloodstream, potentially influencing urinary system function and predisposing individuals to conditions like kidney stones, urinary tract infections, and carcinogenesis [6]. 

The challenge in defining a “core urinary microbiome” stems from a lack of standardized sampling protocols. Studies often include diverse sample populations, combining men and women across a wide age range, and using various methods to collect urine samples [7,8,38,39]; they are also conducted without stratifying for confounding factors such as diet, medication intake, or comorbidities. Despite ongoing debates about sampling differences, it is essential to consider the lower urogenital tract as a gateway to the bladder’s urinary microbiome. Rather than viewing the urethral and bladder microbiomes as separate entities, they should be seen as interconnected due to their lack of physical division [37]. The bladder, with its relatively static features related to urine storage, serves as a reservoir for the urinary microbiome, contrasting with other parts of the urinary tract [40]. This perspective is supported by a study that found no differences in bacterial composition between midstream first-in-the-morning urine samples and catheterized urine samples [41], although evidence remains conflicting [17,40].

The ethiopathogenic role of the urinary microbiome in urothelial cancer has been investigated in recent years. Distinct microbiome signatures have been observed in colon cancer [42]. It is, therefore, also logical to hypothesize that a similar relationship may exist between the urinary microbiome and bladder cancer. We extensively reviewed different studies to summarize the microbiome signature in bladder cancer; only a few studies have provided an extensive analysis of the urinary microenvironment. Studies consistently report no differences in alpha and beta diversity, and report a predominant representation of the phyla *Proteobacteria*, *Firmicutes*, and *Actinobacteria* [9,10,13]; however, at a genus level, *Howardella*, *Streptococcus* [10], *Actimomyces*, *Achromobacteri*, *Brevibacterium* [11], *Anaerococcus*, and *Pseudomonas* [43] were used to distinguish patients with bladder cancer, with *Ralstonia* being used as an additional marker at the species level [13] (Table 3).

The relationship between the urinary microbiome and PD-1 receptor expression has been associated with a pivotal role in treatment response to anti-PD-1 and anti-CTLA-4 agents, providing an important role for microbiota characterization, as PD-L1-positive patients exhibit higher bacterial richness in both alpha and beta diversity compared to PD-L1-negative subjects; they also exhibit a decrease in *Bacteroidetes* at the phylum level and an increase in *Leptotrichia* at the *genus* level [22]. Several studies have also observed associations between the microbiome and anti-PD-1 therapy in various cancers [44]. 

The promising identification of a microbiome signature in patients with bladder cancer represents a significant step toward understanding the connections between urinary microbiome alterations and their implications in oncogenesis, therapeutic strategies, and prediction to treatment responses. This characterization holds potential for tailoring personalized treatments, including chemo-immunotherapy, with the goal of improving oncological outcomes. Early clinical trials administering *Lactobacillus* have shown modifications in the microbiome of NMIBC, potentially reducing recurrence rates [45,46]. Thus, identifying new bacterial profiles with high-end techniques may offer new therapeutic options that are yet to be explored. The potential interactions between the urobiome and BCG therapy may enhance antiproliferative and cytotoxic effects. Furthermore, through the fibronectin–BCG attachment mechanism, a favorable urinary microbiome could regulate and diminish the presence of bacteria binding to fibronectin, thereby enhancing BCG efficacy by increasing available fibronectin binding sites [1]. Many of the studies discussed lack independent validation. Future prospective studies are needed to confirm these findings and support its clinical significance.

## 11. Conclusions

The study of the microbiome is a fertile field of investigation, generating new questions that need exploration. Its potential utility in bladder cancer as both a biomarker and adjunct treatment strategy mirrors observations in other body systems, underscoring the urinary system’s significance in microbiome research. Given the complexity of microbiome data, exploring these paths represents an increasingly ambitious yet rewarding goal.

## Figures and Tables

**Table 1:** Summary of observations on the microbiome in bladder urothelial carcinoma.

Disease Stage	Findings	Methods	Population	Sample Collection	Author
Primary or recurrent NMIBC	No differences in alpha diversity or Simpson index between groups (182 OTUs for NMIBC vs. 184 for patients without bladder cancer). *Firmicutes*, *Actinobacteria*, *Bacteroidetes*, and *Proteobacteria* were the most abundant phyla. Patients with bladder cancer showed enriched OTUs: *Ruminococcaceae* family, *Campylobacter hominis*, *Actinobaculum massiliense*, and *Jonquetella anthropi.*	DNA extraction with PowerSoil^®^ (MoBio Laboratories, Inc. Radnor, PA, USA). DNA quantification via Qubit^®^ (Thermo Fisher Scientific, Waltham, MA, USA) 16S rRNA (V4 region) gene library preparation and MiSeq sequencing (Illumina, Inc. San Diego, CA, USA). Sequenced paired-end reads processing with USEARCH into OTUs. PERMANOVA analysis for risk association. Bladder cancer tissue analysis through *F. nucleatum* PCR.	Total of 23 men: 12 NMIBC and 11 healthy individuals.	Urine specimens. Bladder cancer tissue.	Bučević Popović (2018) [9]
Primary or recurrent bladder cancer	No differences found in the urinary microbiome between bladder cancer group and controls without bladder cancer at the alpha and beta diversity levels. Both groups showed that *Firmicutes*, *Proteobacteria*, and *Actinobacteria* were the most dominant phyla. At the genus level, *Lactobacillus* was more common in patients undergoing BCG therapy. Genera *Howardella* and *Streptococcus anginosus* were more common in female patients.	DNA extraction with QIAamp DNA Micro Kit (QIAGEN, Hilden, Germany). 16s rRNA sequencing (V3 and V4). Sequenced paired-end reads processing with QIAseq (QIAGEN) 16s/ITS kit. Quantitative analysis with QuantiFlour^®^ (Promega, Madison, WI, USA) QIIME2 for bioinformatic analysis.	Total of 25 patients: 18 with bladder cancer and 7 patients without bladder cancer; 73% were men.	Midstream urine sample	Chorbinska (2023) [10]
Bladder cancer: either NMIBC or MIBC. Eleven receiving BCG.	No difference in alpha diversity. Differences in beta diversity at the genus level and phylum levels: Actinobacteria and Proteobacteria showed higher representations in patients with bladder cancer. Phylum level: Firmicutes and Deinococcus-Thermus were more abundant in controls. Genus level: Actinomyces, Achromobacter, Brevibacterium, and Brucella. These were more abundant in bladder cancer. Patients responding to BCG showed no differences in alpha or beta diversity.	DNA extraction with CAT# 47050. DNA sequencing with 16S rRNA of V3 and V4 rDNAs. Analysis in OTUs using QIIME software and polishing with ChimeraSlayer. OTUs were summarized at the Genus level. Assessment of alpha diversity and beta diversity scores using PERNAOVA.	Total of 43 patients with bladder cancer and 10 controls without bladder cancer.	Cases: transurethral. Controls and cases not undergoing a procedure: clean-catch midstream samples.	Hussein (2020) [11]
Bladder cancer comparing NMIBC and MIBC.	Statistically significant differences in alpha and beta diversity between groups. Analysis at the phylum level in both MIBC and NMIBC showed a major representation of Proteobacteria (68.45% and 39.09%), Firmicutes (14.76% and 19.17%), and Actinobacteria (12.98% and 14.92%, respectively). Analysis of differential abundance in MIBC at the phylum level showed abundance of Proteobacteria and decreased Bacteroidetes and Firmicutes. At the species level, Ralstonia was an important marker of MIBC.	DNA extraction with TIANamp MicroDNA kit (TIANGEN Biotech, Beijing, China). Sequencing was processed based on the recognition of Type IIB restriction endonuclease. Cleaned readings were assessed using the 2bRAD-M tags for library identification. Alpha diversity was calculated with the CHao1, Shannon, and Simpson indexes. Beta diversity was assessed with Bray–Curtis, Binary Jaccard, and Euclidean distances.	Total of 22 subjects: 7 with NMIBC and 15 with MIBC.	Bladder tissue	Jian-Xuan (2023) [18]
Three groups: patients without cancer (benign urologic conditions), patients with NMIBC, and patients with MIBC.	Controls showed higher diversity and richness when compared to patients with NMIBC and MIBC, as well as when compared to high-grade and low-grade samples. *Bacteroidetes*, *Lachnoclostridium*, and *Burkolderia* were mostly represented in control samples, while MIBC showed a major representation of *Bacteroides* and *Faecalbacterium*.	DNA isolation with using the ZymoBIOMICS (Zymo Research Corporation, Irvine, CA, USA) DNA/RNA kit. PCR products were analyzed on a 2% agarose E-Gel and products were purified with QIAquick gel extraction kit (QIAGEN). Sequences were analyzed for taxonomy using the silva version 132 classifier. Alpha and beta diversity were measured.	Total of 37 subjects: 10 in noncancer group; 15 in muscle-invasive group; 12 in non-muscle-invasive group.	Midstream samples	Chipollini (2020) [15]
Subjects without bladder cancer compared to patients with bladder cancer, either NMIBC or MIBC.	A consistent representation of five genera was observed: *Streptococcus*, *Bifidobacterium*, *Lactobacillus*, *Veillonella*, and *Actinomyces.* Of these, general *Actinomyces* showed a higher representation in the group with bladder cancer, suggesting a positive correlation with bladder cancer. Within this genus, *A. europaneus* was higher compared to in the control group.	DNA extraction and 16S rDNA sequencing, PCR amplification of V3–V4 region. Sequencing reads were processed using QIIME, from which alpha diversity was calculated. Sequences were organized into OTUs and assigned taxonomy using the Greengenes database.	Total of 55 patients: 29 patients with bladder cancer and 26 controls without bladder cancer.	Midstream clean-catch sample collection	Bi (2019) [12]
Patients with cancer compared to patients without cancer. Patients with cancer included those with NMIBC and MIBC.	Comparison between patients with cancer and controls without bladder cancer at phylum level showed a major representation of *Proteobacteria* (39.7% vs. 49%), *Firmicutes* (32.8%, 28.1%), *Actinobacteria* (7% vs. 6.2%), and *Bacteroidetes* (3.9%, 9.4%); these differences were not statistically significant. At genus level *Acinetobacter*, *Anaerococcus*, and *Rubrobacter* were more represented in patients with cancer.	DNA sequencing with 16s rRNA from V4 regions (Dneasy kit, QIAGEN). OTUs were identified using UPARSE and aligned for a taxonomic analysis using the SILVA and Greengenes databases. Alpha and beta diversity was assessed using QIIME.	Total of 49 patients: 18 without bladder cancer and 31 with bladder cancer (26 with NMIBC and 5 with MIBC).	Midstream clean-catch urine sample collection	Wu (2018) [14]

**Table 2:** Summary of observations on the microbiome in relationship with BCG administration and response.

Disease Stage	Findings	Methods	Population	Sample Collection	Author
Intermediate-/high-risk NMIBC	No differences in microbiome diversity between patients with bladder cancer and controls. No association between bladder microbiota and tumor features. *Lactobacillus*, *Streptococcus*, and *Cutibacterium* were enriched in patients without persistent disease. BCG does not integrate to the bladder microbiota; BCG was observed in only 9% of maintenance samples.	DNA extraction with QI Aamp Kit (QIAGEN, Hilden, Germany). Amplicon sequencing with 16S rRNA. Paired-end reads were processed using QIIME 2, amplicon splice variants were taxonomically classified with Naive Bayes classifiers. Alpha diversity was assessed with the ASV richness, Gini–Simpson, and Shannon indexes. Beta diversity was assessed with ANCOM-BC.	A total of 32 male patients with intermediate-/high-risk NMIBC vs. 41 with benign prostatic hyperplasia.	Catheterized urine samples taken before BCG instillation (induction 1, 5, and 6; maintenance 1) and immediately before TURP for the control group.	Heidrich et al. (2024) [26]
High-grade NMIBC	Significant differences in Shannon and Simpson indexes between patients who experienced recurrence and those who did not. The group that recurred showed more evenness in bacterial representations. Relative abundance of *Aerococcus* was higher in the recurrence group, whereas *Escherichia/Shigella* and *Ureaplasma* were higher for patients who did not experience recurrence.	DNA was extracted and sequenced with 16S rRNA, followed by amplification of the V4 region using Illumina MiSEq primers 515F and 806R (Illumina, Inc. San Diego, CA, USA). Taxonomy was assigned according to the SILVA database. Alpha diversity: evenness was computed with Pielou index; richness and evenness were assessed with the Shannon index; abundance was assessed with the Simpson index.	Total of 29 patients undergoing intravesical therapy for NMIBC: 27 received BCG, 2 patients received intravesical gemcitabine.	Catheterized urine samples prior to first and all six BCG = induction doses. A 7th specimen was obtained before the 3-month cystoscopy.	James et al. (2023) [29]
High-risk patients	Most abundant phyla in both groups were *Actinobacteria*, *Bacteroidetes*, *Firmicutes*, *Proteobacteria*, and *Tenericutes.* An abundance of *Proteobacteria* was observed in patients with recurrence, Lactobacilalles were more abundant in patients with no recurrence.	16s rRNA gene sequencing. Rest of methodology not specified.	Total of 31 patients were enrolled: 10 with recurrence and 21 with no recurrence at 6-month follow-up.	Catheterized urine samples	Sweis (2019) [27]
Various stages of treatment	No significant difference in alpha diversity when comparing patients with cancer vs. controls. Beta diversity was different between groups: *Actinobacteria* and *Proteobacteria* were more abundant in patients with bladder cancer. *Firmicutes* and *Deinococcus* were more abundant in controls. At the genus level, *Actinomyces*, *Achromobacter*, *Brevibacterium*, and *Brucella* were more abundant in patients with bladder cancer. *Salinococcus*, *Escherichia*-*Shigella*, and *Lactobacillus* were more abundant in controls without bladder cancer. In BCG responders, the phyla *Firmicutes* and *Proteobacteria* were more abundant. At the genus level, *Serratia*, *Escherichia-Shigella*, and *Pseudomonas* were more abundant in the responders.	DNA extraction and sequencing with 16S rRNA. Paired-end reads were analyzed with QIIME, and OTUs were referenced with the SILVA database. Measurement of alpha diversity was conducted with the Chao1, Shannon, and Simpson indexes. Beta diversity was measured with the Bray–Curtis score and PERMANOVA.	Total of 43 samples from patients with bladder cancer and 10 samples from controls without bladder cancer.	Catheterized urine samples from patients with bladder cancer. Clean-catch midstream samples obtained from volunteers without bladder cancer and patients with bladder cancer that were not undergoing a procedure.	Hussein (2020) [11]

**Table 3:** Summary of microbiome composition according to patient features.

Sample of Study	Microbiome Fingerprint	References
Men without bladder cancer	Phyla: *Firmicutes*, *Actinobacteria*, *Fusobacteria*, *Proteobacteria*, *Bacteroidetes.* Genus: *Lactobacillus*, *Corynebacterium*, *streptococcus.*	Nelson (2010) [4]
Women without bladder cancer	Phyla: *Firmicutes*, *Bacteroidetes*, *Actinobacteria*, *Fusobacteria.* Genus: *Lactobacillus*, *Prevotella*, *Gardnerella.*	Siddiqui (2011) [5]
Bladder cancer	Phyla: *Firmicutes*, *Proteobacteria*, and *Actinobacteria*, *Bacteroidetes.* OTUs: *Fusobacteria*, *Actinobacteria*, *Facklamia* and *Campilobacter.* Strain level: *Campylobacter hominis*, *Actinobaculum massiliense*, and *Jonquetella antrophi.* Species: *Fusobacterium nucleatum.*	Bučević Popović (2018) [9], Chorbińska (2023) [10], Hussein (2021) [11], Bi (2021) [12], Sun (2023) [13], Wu (2018) [14], Chipollini (2020) [15]
PD-1-positive	*Phylum: Fusobacteria*, *Proteobacteria. Genus*: *Actinomyces*, *and Leptotrichia.*	Chen (2022) [22]
BCG responders	*Genus: Lactobacillus*, *Streptococcus and Cutibacterium.* Revisar: *Escherichia*/*Shigella*, *Ureaplasma*	James (2023) [29], Sweis (2019) [27], Heidrich (2024) [26]
Virome	HCMV, EBV, HHV-6, HPV, BKPyV, TTV.	Hrbáček (2023) [31]
